# Rice the Cause of Epidemic Cholera!

**Published:** 1834

**Authors:** 


14. *Rice the cause of Epidemic Cholera!* We are happy to learn by the journals, that a Dr. Tytler, a "graduate of the University of Edinburgh and a surgeon in the service of the East India Company," has at length discovered the cause of epidemic Cholera, which proves to be nothing more nor less than Rice—diseased rice of course, or it would not excite the Cholera. It was reserved for the Medical Society of London, to be the recipients of this important discovery; and it must have been edifying, to observe the members of that learned association, night after night, engaged in discussing the merits of this new revelation on the cause of Cholera. To us, who live on the banks of the Ohio, instead of the Thames, and grope our way in the woods, the whole matter smacks somewhat of the diverting; but this arises no doubt from not understanding what ought to occupy the time of an assembly of *London* doctors.

We shall not undertake to lay before our readers, the various facts on which this sapient "graduate of the University of Edinburgh," rests his hypothesis; but would advise our readers to rely, as we ourselves do, on what all the world knows, that the discharges in Cholera are rice-water, which, of course, they could not be, if rice had not been eaten. This fact of itself would seem to be sufficient; but if it is not, we fear the doctor's hypothesis will fall to the ground. If any one should ask how it happens, that persons who never taste rice get the Cholera; why it becomes epidemic at the same time over extensive tracts of country, suddenly appearing and disappearing, while the consumption of rice remains stationary; or why the disease has broken out *progressively* from India to Mexico, we must refer them to Dr. Tytler and his partizans in the London Society, who no doubt would be pleased to clear up their difficulties and dissipate their incredulity.

